# Adapting Standards: Ethical Oversight of Participant-Led Health Research

**DOI:** 10.1371/journal.pmed.1001402

**Published:** 2013-03-12

**Authors:** Effy Vayena, John Tasioulas

**Affiliations:** 1 Institute of Biomedical Ethics, University of Zurich, Zurich, Switzerland; 2 Faculty of Laws, University College London, London, United Kingdom; PLoS Medicine, Canada

## Abstract

As participant-led health research increases, Effy Vayena and and John Tasioulas examine what ethical questions are raised, and what types of standards need to be developed for appropriate ethical oversight for participant-led research projects.

**Summary Points**

- Online social media and digital technologies have facilitated formation of communities of individuals engaged in establishing and conducting health research projects. The results of such participant-led research (PLR) have already appeared in leading biomedical journals.
- These projects involve research with human participants. Hence, what are the requirements for ethical oversight? To what extent is standard ethics review also suitable for PLR?
- A comparison of PLR with standard research reveals six areas that are of potential relevance to ethical oversight: institutionalization, state recognition and support, incentive structures, openness, bottom-up approach, and self-experimentation.
- The distinctive nature of PLR requires adaptation of ethical oversight standards to the character of such research. These should strike a balance between protecting interests of research participants and achieving promised benefits of PLR.
- The appropriate form of ethical oversight for PLR projects depends on which of three categories they fall into. If they meet the “institution-plus” criterion, standard ethics review applies. If not, then the appropriate form of oversight depends on the application of a minimal risk criterion.

## Introduction

Increasing access to digital technologies and proliferation of online social networks have enabled individuals to become more active in regulating their personal health [1],[2]. These trends have also facilitated the formation of communities of individuals engaged in establishing and pursuing health research projects [3],[4]. The type of research conducted by these communities includes self-experimentation, self-surveillance, analyses of genomic data, and genome-wide association studies (GWAS) [5]. These projects are described as “citizen driven” [6], “participant driven” [7], “crowd sourced” [8], or “participant centric” research [9],[10]. What they have in common is that participants are the leading force in the initiation or conduct of research projects. Hence we refer to such projects as participant-led research (PLR).

Recently, results of several PLR initiatives have appeared in high-impact scientific journals [11]–[13]. In 2011 Nature Biotechnology published a study of the effects of lithium on the progression of amyotrophic lateral sclerosis. This study arose from self-experimentation by a group of participants on a website called patientslikeme.com. Researchers from patientslikeme.com provided algorithms to match controls, undertook data analysis, and wrote up the results for publication [14]. The findings were subsequently confirmed by clinical trials. There are numerous examples of PLR, which differ by subject matter and methodology. One example is the “butter-mind study experiment” undertaken by 65 people on Genomera, a web portal for group health studies, to determine the effects of fat intake on performance of mathematical calculations [15]. The study results are available online. Another example is the 23andMe inVite study of genetic influences on the response to treating metastatic breast cancer with bevacizumab [16].

PLR, whilst potentially a boon to research [17] and the P4 approach to medicine—medicine which is personalised, predictive, preventive, and participatory—poses a number of challenges [18]. These revolve around two questions. Can PLR achieve the scientific rigor needed to complement standard health research? And, if so, how can it be conducted ethically? Here, we focus on the latter question. In particular, on whether adequate ethical oversight of PLR must involve standard ethics review.

The principal justification for ethical oversight in research with humans is protection of research participants. In the case of standard research, an institutional review board or ethics review committee is responsible for assessing a number of factors, including risk/benefit ratio, quality of informed consent procedures and materials, competence of researchers, and compliance with obligations to participants. International guidelines and national legislation require investigators to obtain ethics approval before conducting their studies [19],[20]. Should people engaged in PLR comply with the standard ethics review process? People considering involvement in PLR projects, editors of scientific journals [21], as well as the public with whom the PLR movement is seeking to build credibility, require guidance on these matters.

Unfortunately, there is no sustained attempt in the literature to address this issue [22]. Some attention has been given to informed consent in PLR, but it is doubtful that ensuring participants receive appropriate information is sufficient ethical oversight [23]. In this paper we approach the ethical oversight question by first comparing PLR with standard research to identify the extent to which similar ethical concerns are raised. On the basis of this comparison we make a proposal for adapting current ethical oversight standards to address the distinctive concerns raised by PLR.

## Caveats

We start with the following caveats:

First, we assume that PLR is, in principle, capable of achieving the level of scientific rigor required to warrant publication of its findings in reputable scientific journals. This assumption is contestable in light of the methodological limitations of PLR, including bias, self-selection, and problems with self-reporting of symptoms or phenotypic data [8],[24]. Moreover, if a proposed study fails to meet requisite scientific standards, it is likely to be ethically impermissible to pursue it [18].

Second, our concern here is the moral obligation regarding ethical oversight that arises in the case of PLR. It is a separate question to what extent this obligation is properly made socially enforceable, whether through law or some other mechanism.

Third, we assume that the standard model of ethics review is broadly appropriate for standard research. Ethics review aims to protect research participants from unreasonable risks, coercion, exploitation, and unfair distribution of burdens and benefits. It bears noting, however, that the existing practice of research ethics review has been criticized as unduly burdensome, paternalistic, and capable of costing patients' lives [25]–[27].

Fourth, PLR is a heterogeneous phenomenon and the lines between it and standard research are often blurred. For example, the GWAS conducted by 23andMe (a direct-to-consumer genomic provider with a research arm) were described as “participant-driven” [5]. However, they were initiated by 23andMe investigators, with participants being called upon to contribute by filling out various surveys. Participants had a choice whether or not to fill out a survey, but such “participant-driven” studies closely resemble those initiated by investigators.

## A Comparison of Participant-Led with Standard Research

**Figure 1:**
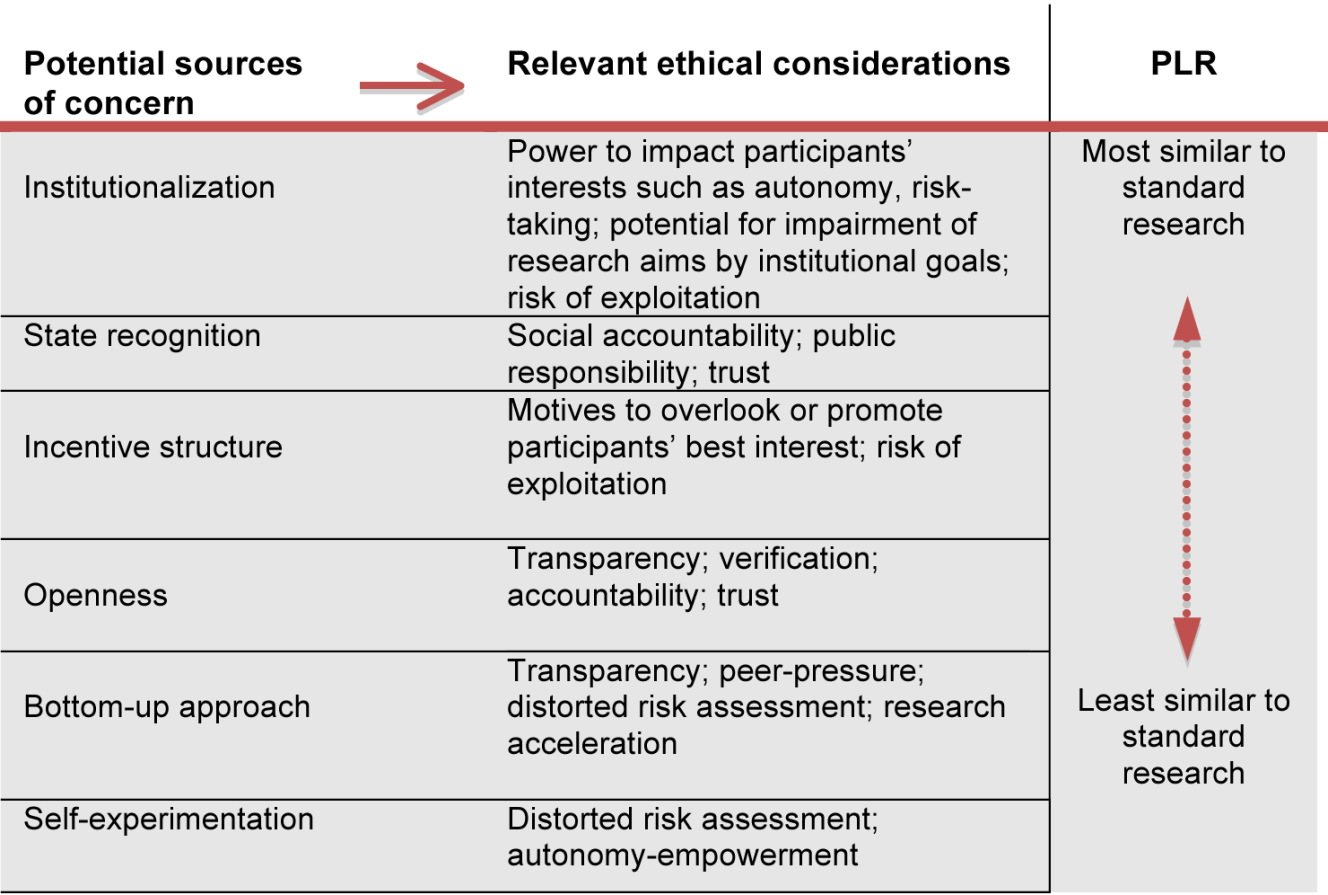
Ethical considerations in PLR (resulting from comparing PLR with standard research).

Comparing PLR and standard research reveals at least six areas that are important with regard to obligations of ethical oversight. Figure 1 summarizes these sources of concern and the ethical considerations they generate.

### Institutionalization

Both types of research aim at producing generalizable health knowledge. The primary agents in standard research typically belong to standing institutions, rather than being members of informal groups of individuals that come together to pursue a one-off project. These institutions involve hierarchies of authority, often backed up by legal or other sanctions, and may have access to considerable resources. As a result, institutional agents generally have a greater capacity to affect the interests of individuals, e.g., to coerce them into involvement in research. In addition, institutional structures may prioritize some institutional goal (e.g., profit-making, reputation building, etc.) that can obstruct the pursuit of valuable research [28],[29]. In such cases, a worrying mismatch may arise between the motives of the participants (for example, altruism or interest in contributing to the advancement of knowledge) and the goals of the researchers, leading to the possibility of participants being exploited or misled.

Admittedly, some forms of PLR also exhibit an institutional dimension. For example, a for-profit company like 23andMe clearly counts as an institution, and similar not-for-profit outfits may also do so. Conversely, in forms of PLR that lack a clear institutional structure, the responsibilities of participants may be ill-defined, and could result in failure to integrate ethical considerations into decision making.

### State Recognition

The institutions that carry out standard research are typically recipients of official state recognition (e.g., universities or liability limited research companies) and, often, the beneficiaries of material public support, (e.g., research grants, tax benefits, subsidies, etc.). This research enterprise is accountable to the public, in whose name recognition and support is given, and a key concern of such accountability is compliance with ethical standards. The public, in turn, bears a special responsibility to hold state-supported and recognized bodies accountable in this way. Moreover, the public imprimatur conferred on such institutions generates a responsibility not to abuse the trust thereby bestowed. On the other hand, institutions that have received state recognition and support have usually been officially vetted in some way, e.g., as meeting ethical and other requirements applicable to operating as a university. Again, to the extent that the agents pursuing PLR are state-recognized and supported, e.g., as legally registered corporations, the preceding points will apply to them.

### Incentive Structures

Researchers may be motivated not only by the goal of advancing medical knowledge, but also by profit-making, career-advancement, impacting policy, etc. This can create incentives to infringe ethical requirements applying to research, including those governing risk of harm and non-exploitation. Yet, PLR is hardly free of incentives to engage in unethical behavior. For example, some PLR takes place within a profit-making structure. And there are also potentially distinctive incentive issues within PLR. Given that those conducting the research often hope to benefit personally from its outcome (e.g., in experimenting with an off-label use of a medication), they may be led to engage in unacceptable forms of risk-taking, and to pressurizing others to follow suit.

### Bottom-up Approach

Some forms of PLR arise out of a shared interest among a group of non-experts, e.g., those who suffer from a rare disease. By contrast, standard research is typically driven by the interests and agenda of the established scientific community. As a result, PLR is not only potentially an exercise of personal autonomy and empowerment on the part of those involved, it is also an avenue for pursuing research into topics that are overlooked or sidelined by the scientific establishment. Moreover, the flexibility of the bottom-up approach can lead to an accelerated pace of research. Yet, given how group dynamics may develop, a concern that arises here is the inappropriate use of peer-pressure to promote participation in a research project. An additional source of concern arises when studies are carried out by individuals who lack research credentials.

### Openness

PLR characteristically exhibits openness in its workings. Study protocols and design are accessible to participants, e.g., in crowd-sourced research. They are often posted online and are accessible to a wider community, even if not to absolutely everyone. Openness facilitates transparency, which is a general ethical demand on any social decision-making process affecting others. Despite transparency requirements such as audits for publicly recognized and supported institutions, standard health research is widely considered to have fallen short of fulfilling its obligation of transparency to society. A vivid illustration is the general failure to report negative results from clinical trials. Even after the introduction of the clinical trial registration system, concerns persist about the selective publication of positive results and the consequent distorting effects on the assessment of drug effectiveness and prescription recommendations [30].

### Self-Experimentation

Some forms of PLR involve self-experimentation, e.g., by an intervention study involving over-the-counter drugs, dietary supplements, or prescription drugs. Other forms involve the use of genetic information or other personal health data. By contrast, standard health research seldom involves self-experimentation on the part of the investigators. Self-experimentation has a bearing on the reliability of risk-benefit analyses. First, researchers may generally run greater risks of harm when they are not themselves liable to incur it [31]. Second, when participants in PLR have a personal stake in the research outcome (e.g., terminally ill with no available treatment), they may be led to undertake unacceptable risks [32]. Finally, there is a general difference of principle: regulation of standard health research with human participants is primarily based on a concern not to harm others, whereas regulation of PLR involving self-experimentation largely reflects paternalistic considerations—preventing harm to self. Generally speaking, respect for individual autonomy means that harm to others is a more robust basis for founding a moral obligation than harm to self, especially where the obligation is socially enforceable.

The above comparison reveals that participants in PLR initiatives may also be at risk of harm and therefore ethical oversight ensuring appropriate protections is needed. However, the differences between standard health research and PLR have complex implications for what constitutes appropriate ethical oversight of PLR. Balancing these considerations is further complicated by the diverse nature of PLR. In light of this complexity, it is unlikely that a single formulation of the obligation of PLR participants regarding ethical oversight strikes a uniquely correct balance of the various considerations. However, it is still worth searching for a formulation that is plausible, adapted to the distinctive character of PLR, capable of attracting wide consensus, and that can be operationalized without excessive difficulty. In order to stimulate debate on the appropriate form of ethical oversight for PLR, we propose the following approach.

## Adapting Ethical Oversight to Participant-Led Research

We propose that the appropriate form of ethical oversight for PLR depends on which of the following three categories any given project falls into.

### Category I

Here we place those forms of PLR that are subject to the standard form of ethics review that is also applicable to standard research. PLR belongs to this category if it is carried out by agents that satisfy the “institution-plus” criterion, i.e., they are institutions that are either state-recognized, even if not state-supported, or are engaged in profit-making. The “institution-plus” criterion picks out those forms of PLR that are the same as standard research for the purposes of ethical oversight, and hence are subject to identical obligations of oversight, i.e., standard ethics review.

For those forms of PLR that do not meet the “institution-plus” criterion, we propose two further categories. We suggest a risk-based approach based on applying the minimal risk criterion, i.e., risks attached to routine medical and psychological examination [19]. Such risks include not only physical harm, but also psychological and social harm, including privacy violation. In standard ethics review, the ethics review committee makes the decision to conduct expedited review or allow exemption from review, on the basis of the minimal risk criterion. In the case of PLR it can be made by the participants in the project, or a designated group within the project that focuses on participant protection.

### Category II

If the research involves more than minimal risk to participants, then some form of ethics review is required. One possibility may be the creation of forms of ethics review that are equivalent to expedited review. For example, “citizen ethicists” have been proposed as analogous to citizen scientists. Another proposal worth considering is an open protocol review that uses crowd-sourcing for ethics review [33]. A possible outcome of such expedited review is a recommendation that standard ethics review be carried out. This might be, for example, when the risk to participants reaches a certain threshold.

### Category III

**Figure 2:**
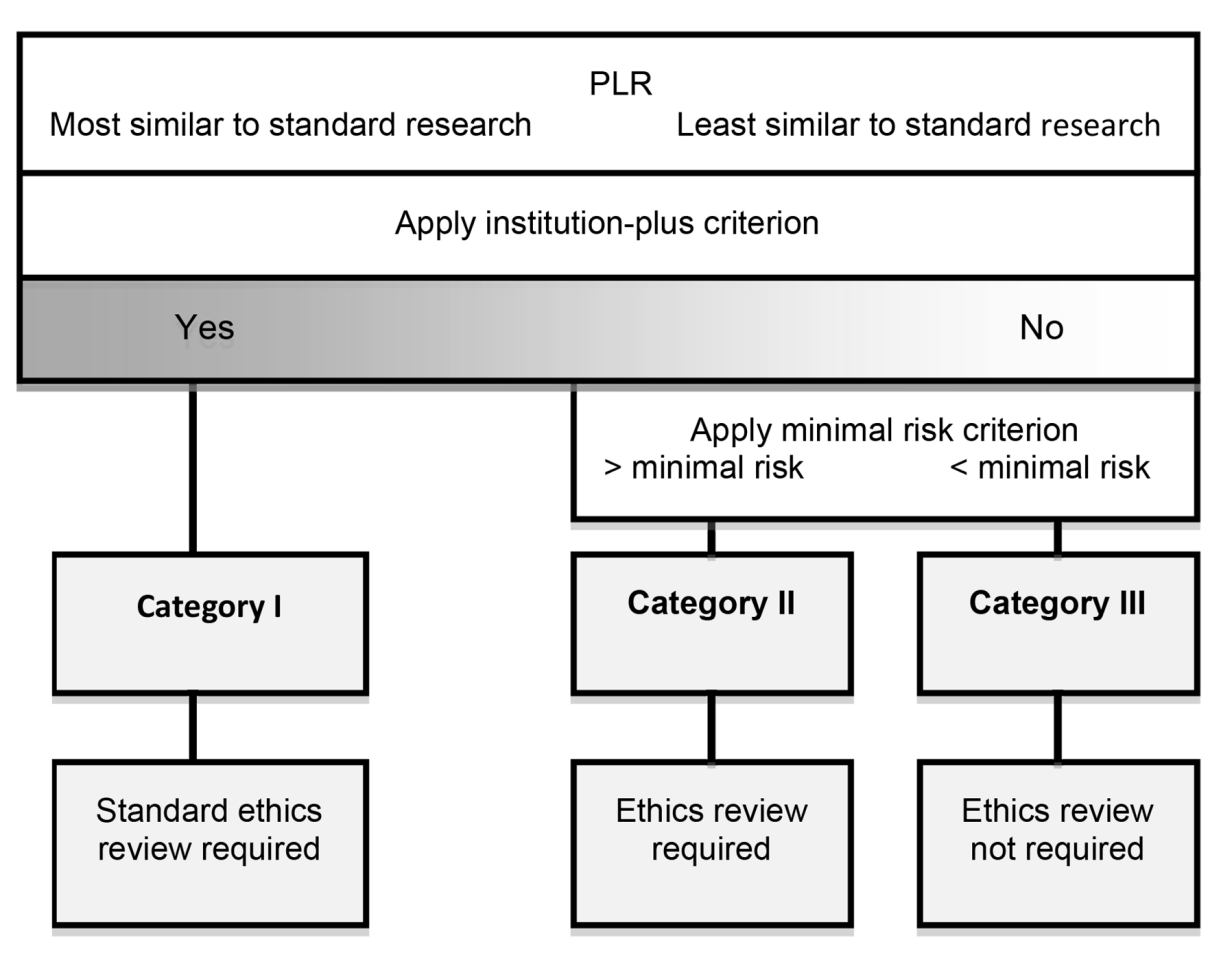
A proposal for ethical oversight of PLR.

If the proposed research involves no more than a minimal risk then no additional formal ethics review is morally required. A well-grounded finding of minimal risk, although it exempts a PLR project from formal review, obviously does not exempt those engaged in it from exercising the level of ethical oversight we properly expect of people in day-to-day life. This includes considering respect for autonomy, avoiding unjustified risk of harm, fair distribution of burdens and benefits, and due respect for the law. Figure 2 summarizes the proposal schematically.

We believe that this scheme merits further discussion as one way of striking an appropriate balance between protecting the interests of research participants and realizing the distinctive benefits of PLR. In this way, we might prevent ethics review becoming a strait-jacket on PLR-inspired innovation, stifling individual liberty, and serving as a disincentive to non-experts who might otherwise make valuable contributions to medical knowledge. This proposal leaves open the issue of enforceability, which also requires further debate.

## Conclusion

PLR holds out the alluring prospect of citizen engagement in the co-production of knowledge with the scientific community [34],[35]. But like any form of scientific research involving human participants it is subject to ethical as well as scientific standards of appraisal. The appropriate standards of ethical oversight for PLR need to be adapted to its distinctive nature. This article is a contribution to a much needed broad-ranging dialogue that engages various stakeholders. Failure to adequately address this issue, and to generate consensus on best practice, poses a threat of harm to participants, risks undermining the credibility of PLR, and may eventually provoke a backlash of over-regulation that deprives us of its potential benefits.

## Abbreviations

PLR: participant-led research
